# Fœtal Deformities

**Published:** 1874-05

**Authors:** J. A. Meek

**Affiliations:** Jonesboro, Ark.


### FŒTAL DEFORMITIES.

BY J. A. MEEK, M.D., JONESBORO, ARK.

It is with no inconsiderable degree of trepidation that I make my debut upon the arena of Medical Literature.

This trepidation arises from a two-fold cause: First, because that my isolated field of practice gives no advantages of frequent conversations or interchange of opinions with the learned of our profession. And secondly, because I am not certain that the subject matter of my article has not been previously discussed and elaborated in some medical work or scientific paper yet unnoticed by myself. Without additional circumlocution, I shall proceed to state the object of this communication, hoping it may meet with a kindly reception at the hands of my professional brethren, hailing as it does from the cane-brakes of Arkansas. Its object is to offer a few suggestions upon the subject of fœtal malformation and its causes.

We often witness children born in various stages of development, some have no feet, others no hands. In a word, fœtal developments are to be met with in all stages, from a mole up to a well formed fœtus. All writers upon this subject, which I have had an opportunity of examining, merely say that these malformations are deviations from the natural laws governing fœtal formation. They do not attempt to show how these malformations are produced, or explain the causes which produce these freaks of nature. If our theory of conception be true, then the causes which produce these results are easily explainable. That theory is, if I correctly understand it, that the semen of the male contains numbers of spermatozoa, and that when one of these little spermatozoa enters an ovum of the female and a fixation of the fecundated ovum takes place that conception is accomplished. Perhaps it would be more clearly expressed and better understood by the reader, to adopt the exact language of Prof. Meigs, who is certainly very high authority on this or any other subject about which he has written. He says that "conception is the affixation of a fecundated ovum upon the living surface of the mother." A fecundated ovum is one which contains a spermatozoon. By examination of the spermatozoa with a microscope, we find them in various physical conditions; some are perfectly formed and very active, others seem to be in a crippled or injured state, some seem to have lost a portion of their tails, others have these little appendages broken or curled up, and yet appear lively and active. These injuries we deem the result of of accident, or, to be more explicit, we would say that the spermatozoon has been injured or mangled by some mechanical force acting upon it in some way. When the conception is with a normal healthy spermatozoon the fœtus will be normal and healthy. If the ovum is fecundated by a crippled spermatozoon it will develop into a malformed fœtus. My theory of malformation, in short, is that the little spermatozoon after receiving its injuries may continue to press forward in the fluids of the vaginia and the uterus until it reaches the ovum which it enters and fecundates. In a word I believe and contend that the normal spermatozoon contains all the elements necessary to make a perfect fœtus, which simply develops in the ovum.

If my theory as to the cause and origin of fœtal malformation be correct, it would necessarily follow that the malformation of the fœtus must correspond in locality to that part of the spermatozoon injured before reaching the ovum.

To be more explicit, I contend that the spermatozoon is a fœtus or a perfectly formed child in an embryo state; that is, its organism is perfect, yet so minute that all of its members are not brought to view even under the best miscroscope. Under favouring circumstances this little animal grows or becomes fully developed. The ovum contains all the elements necessary to effect its growth. Before the spermatozoon enters the ovum it is as perfectly formed as it is after birth, although so delicate and tender as to be incapable of independant existence. The development, which takes place in the ovum, qualifies it for a more hardy existence; qualifies it for living in the world in our present state of existence. My object is simply to present my views without argument, reserving my arguments in support of the doctrine until it shall have been attacked.

